# An Evolutionarily Conserved Role for the Aryl Hydrocarbon Receptor in the Regulation of Movement

**DOI:** 10.1371/journal.pgen.1004673

**Published:** 2014-09-25

**Authors:** Evan G. Williams, Laurent Mouchiroud, Michael Frochaux, Ashutosh Pandey, Pénélope A. Andreux, Bart Deplancke, Johan Auwerx

**Affiliations:** 1 Laboratory of Integrative and Systems Physiology, École Polytechnique Fédérale de Lausanne, Lausanne, Switzerland; 2 Laboratory of Systems Biology and Genetics, École Polytechnique Fédérale de Lausanne, Lausanne, Switzerland; 3 Center for Integrative and Translational Genomics, University of Tennessee Health Science Center, Memphis, Tennessee, United States of America; Stanford University School of Medicine, United States of America

## Abstract

The BXD genetic reference population is a recombinant inbred panel descended from crosses between the C57BL/6 (B6) and DBA/2 (D2) strains of mice, which segregate for about 5 million sequence variants. Recently, some of these variants have been established with effects on general metabolic phenotypes such as glucose response and bone strength. Here we phenotype 43 BXD strains and observe they have large variation (∼5-fold) in their spontaneous activity during waking hours. QTL analyses indicate that ∼40% of this variance is attributable to a narrow locus containing the aryl hydrocarbon receptor (*Ahr*), a basic helix-loop-helix transcription factor with well-established roles in development and xenobiotic metabolism. Strains with the D2 allele of *Ahr* have reduced gene expression compared to those with the B6 allele, and have significantly higher spontaneous activity. This effect was also observed in B6 mice with a congenic D2 *Ahr* interval, and in B6 mice with a humanized *AHR* allele which, like the D2 allele, is expressed much less and has less enzymatic activity than the B6 allele. *Ahr* is highly conserved in invertebrates, and strikingly inhibition of its orthologs in *D. melanogaster* and *C. elegans* (*spineless* and *ahr-1*) leads to marked increases in basal activity. In mammals, *Ahr* has numerous ligands, but most are either non-selective (e.g. resveratrol) or highly toxic (e.g., 2,3,7,8-tetrachlorodibenzo-p-dioxin (TCDD)). Thus, we chose to examine a major environmental influence—long term feeding with high fat diet (HFD)—to see if the effects of *Ahr* are dependent on major metabolic differences. Interestingly, while HFD robustly halved movement across all strains, the QTL position and effects of *Ahr* remained unchanged, indicating that the effects are independent. The highly consistent effects of *Ahr* on movement indicate that changes in its constitutive activity have a role on spontaneous movement and may influence human behavior.

## Author Summary

Using 43 strains from the BXD mouse reference population, we observed a 5-fold difference in spontaneous activity. QTL analysis indicated that ∼40% of this variance is due to the aryl hydrocarbon receptor (*Ahr*). *Ahr* is a conserved transcription factor found in nearly all multicellular organisms and implicated in a multitude of functions, ranging across development, liver metabolism, and neuronal health. This gene is highly variant in the BXDs, and strains with the low-active *Ahr* allele have significantly higher voluntary locomotion. This increase is also observed in independent mouse models, which have reduced *Ahr* activity, including in transgenic mice with humanized *AHR*. Furthermore, decreasing *Ahr* expression in *C. elegans* and *Drosophila* causes similar, robust increases in spontaneous movement. This link is independent of major environmental perturbations as well: BXD strains fed high fat diet long-term move only half as much as their chow-fed brethren, yet the effects of *Ahr* were consistent and equally strong in both dietary cohorts. While *Ahr* is a highly liganded transcription factor in mammals, these data indicate that modifications to its constitutive activity are sufficient to control movement. However, certain ligands may be able to specifically act on this phenotypic aspect of the gene.

## Introduction

Recent studies have highlighted the utility of the BXD murine genetic reference population in the study of metabolism [1]. The BXD population consists of ∼100 related strains descended from C57BL/6J (B6) and DBA/2J (D2) [2], and has wide phenotypic variance in key traits such as blood pressure [3], body weight, and glucose response [1] caused by ∼5 million sequence variants segregating across the population. Many specific variants have been established as causal of overt phenotypic changes, including SNPs in the aryl hydrocarbon receptor (*Ahr*) mediating TCDD response [4], missense SNPs in alkaline phosphatase causing impaired vitamin B_6_ metabolism and bone weakness [1], and a CNV of glyoxalase 1 causing increased anxiety [5]. However, the full phenotypic consequences of such variants in the BXDs are only partly understood.

*Ahr* is a basic helix-loop-helix (bHLH) transcription factor that has been established over the past decades as a key regulator of a variety of processes, including embryonic development [6], xenobiotic metabolism [7], immune response and inflammation [8], and tumorigenesis [9]. In its inactive form, the AHR protein is localized in the cytoplasm with a variety of chaperones, such as HSP90. When activated, AHR dissociates from its chaperones, dimerizes with the aryl hydrocarbon nuclear translocator (ARNT) [10], and translocates to the nucleus. This complex then induces the transcription of a multitude of target genes [11]. In vertebrates, AHR is constitutively active but can also be activated by endogenous ligands such as kynurenine and dietary compounds such as indirubin [8], [12]. These ligands can induce diverse transcriptional networks leading to distinct phenotypic outcomes. For example, indirubin and 2,3,7,8-tetrachlorodibenzo-*p*-dioxin (TCDD) are both high-affinity AHR ligands [13], yet the former is a anti-cancer compound, while the latter is a potent toxin [14], [15]. Natural variants in *Ahr* are also known to strongly affect the activity of AHR within and across species [16], [17], such as in mice, where C57BL/6J (B6) and DBA/2J (D2), the parents of the BXDs, have a ∼20-fold difference in AHR activity due to several missense SNPs [18].

Through the systematic measurement of a large population of BXD mice, we observed large variations in spontaneous activity, a large portion of which was attributable to a single genetic locus on chromosome 12. With the aid of genomic and transcriptomic data, we inferred *Ahr* as the most likely candidate gene responsible for this effect. Through a cross-species approach, from humanized *AHR* mice, to heterozygotic *Drosophila melanogaster* mutants and *Caenorhabditis elegans* exposed to RNAi, we were able to conclude both that *Ahr* is the quantitative trait gene (QTG) and that in each case, reducing its expression increases movement. Subsequently, we examined the effects of high fat diet (HFD) in the BXDs and establish that, while it robustly decreases movement and increases weight, the effects of *Ahr* remain strong and entirely independent. Together, these data indicate a striking new moonlighting phenotypic role for *Ahr* in the control of locomotor activity.

## Results

### Identification of a QTL Influencing Movement

**Figure 1:**
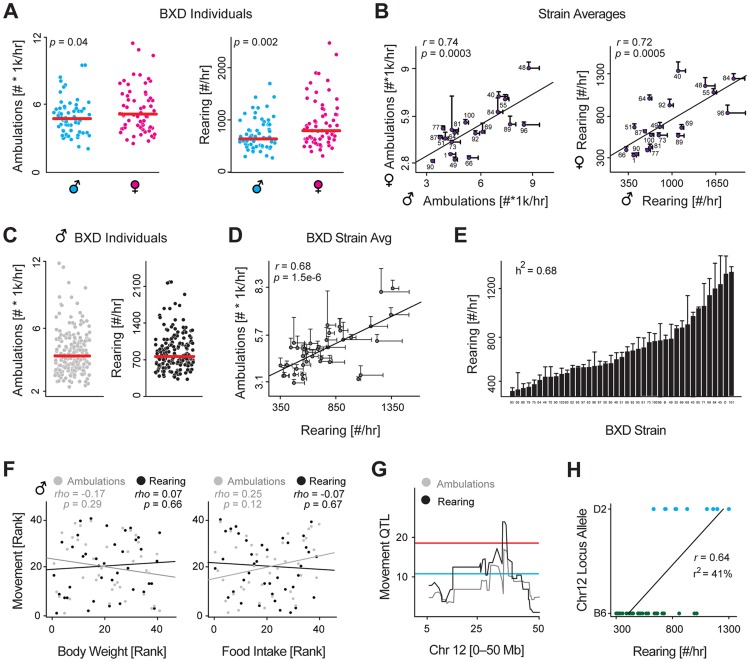
Identification and validation of a movement QTL. (**A**) Nighttime rearing and ambulatory activity for all 68 males and 68 females phenotyped across 22 (male) and 19 (female) strains. These 19 strains were phenotyped in both sexes. Females are slightly more active. (**B**) Despite moving somewhat more, female and male activity are strongly correlated by strain. (**C**) Nighttime rearing and ambulatory activities for all 196 animals across 43 strains. Each strain has ∼5 biological replicates. (**D**) Ambulatory and rearing activity are tightly correlated, though the measurements are technically independent. (**E**) Nighttime rearing activity for all 43 strains, ordered by value. Activity varies by 3.9 fold across the population. The strong heritability (h^2^) of 0.68 indicates that the majority of this variance can be attributed to genetic factors. (**F**) Body weight (Left) and food intake (Right) have no effect on ambulatory or rearing activity, suggesting movement is largely independent of the weight or the need to eat or drink. Animals must rear to reach the food basket or drink. (**G**) Rearing and ambulatory movement mapped to a common narrow 2 Mb locus on chromosome 12. (**H**) The target locus (chromosome 12 from 35.5–37.6 Mb) explains ∼40% of variance (r^2^) in rearing activity and ∼25% of variance in ambulatory activity.

We established a colony of 43 BXD strains and designed a basic phenotyping program to examine how two basic metabolic parameters—activity and weight—vary, and how much of this may be attributed to genetic variants in the population. In a previous study, we examined how many metabolic traits vary due to sex in the BXDs [1], but did not examine basal movement. We thus examined 68 female and 68 male retired breeders from 22 BXDs strains at approximately 20 weeks of age. Animals were placed individually in normal housing cages and spontaneous movement recording over a 48 hour period. Females were slightly more active (Figure 1A), and movement was highly variable for both sexes (∼3-fold for ambulations and ∼4-fold for rearing), but overall, movement was strongly correlated by strain between males and females (Figure 1B), with the range of across genotypes (∼5-fold range) dramatically outweighing the range across the sexes (∼1.3-fold).

To search for the genetic drivers of movement, we designed an enlarged phenotyping program to examine activity in male BXDs using a larger population sample: 43 strains with 5 animals per cohort phenotyped at precisely 23 weeks of age, using the same diet and recording setup. In the expanded data, both rearing and ambulatory activity again varied dramatically across the population—5-fold and 8-fold respectively (Figure 1C). The two aspects of movement were tightly correlated by strain (Figure 1D) and highly consistent for all five biological replicates within each strain, yielding high estimates of heritability (h^2^ = 0.59 for ambulations, and h^2^ = 0.68 for rearing, shown in Figure 1E). The BXD population likewise had highly variable body weights across the population (∼2-fold range) with a high estimate of heritability (h^2^ = 0.74). Surprisingly however, spontaneous activity had no association with body weight or food intake (Figure 1F), indicating the strains' movement is driven primarily by internal motivating factors, rather than by access to food or water, both of which require rearing to reach. Due to the strong heritability and wide and consistent cross-strain variance, we suspected that the movement variation may be linked to quantitative trait loci (QTLs), which could indicate the region(s) of the genome causing the genotypically-driven effects. For both measurements, rearing and ambulation, we detected overlapping QTLs, with the narrow-sense peak located on chromosome 12 from 35.5 to 37.6 Mb, and the broad-sense peak QTL from 30.3 to 37.6 Mb (Figure 1G). For ambulatory activity, this locus explains 25% of the overall variance, or 1300 counts/hr, and for rearing the same locus explains 41% of the variance, or 400 counts/hr (Figure 1H).

**Figure 2:**
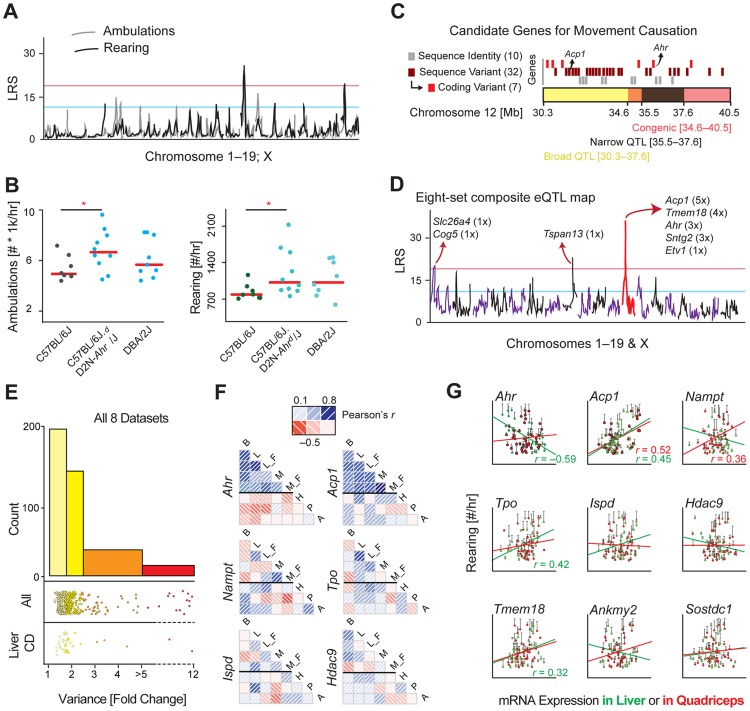
Identification of the movement QTG: *Ahr*. (**A**) The movement QTLs across all chromosomes indicates one consistently significant locus on chromosome 12, and one significant locus on chromosome 19 only for rearing activity. (**B**) A congenic strain on a B6 background with a D2 congenic interval at the region of the chromosome 12 QTL has significantly more spontaneous movement, validating that this locus impacts movement. One-way ANOVA is suggestive (*p* = 0.09), while the *t*-test between only the congenic and control groups is significant (*p* = 0.01). (**C**) Close-up of the congenic (red) and overlapping significant QTL (black) and suggestive (yellow) regions on chromosome 12. All genes are represented by rectangles at their approximate chromosomal positions. Genes that are sequence identical across the BXDs are marked in grey and were discounted. Genes with sequence variants of unknown effect (maroon) and genes with sequence variants with clear effects (e.g. coding differences, red) were considered more likely candidate QTGs. (**D**) eQTLs were plotted for all positional candidates in all eight datasets tissues. Five genes map to chromosome 12 (∼25–45 Mb) as *cis*-eQTLs. As the movement QTL also maps to this locus, *trans*-eQTLs are of less interest. (**E**) Transcript variance was checked across all 9 datasets. Half of the transcripts are at least moderately variable (range >1.75 fold) across the cohorts, while 9% of transcripts are highly variable (>3.0 fold). Upper stripchart: Variance of all 42 candidate genes in all 8 tissues (i.e. each gene is represented ∼8 times). Lower stripchart: Variance of all 42 candidate genes in CD liver. (**F**) Few transcripts are consistently expressed across strains in different tissues (Brown Adipose: B; Liver: L; Muscle: M; Hypothalamus: H, Pituitary: P, Adrenal: A; F: High fat fed cohorts; all others are chow fed). *Ahr* covaries well for all five datasets taken from this study, but negatively with the three publicly available BXD datasets; *Acp1* covaries positively in all datasets, although much stronger in the five paired sets. Most other genes (e.g. the four shown) have little consistency. (**G**) Gene expression correlates with movement inconsistently by tissue, with only a handful of genes yielding consistent (*Acp1, Tpo, Tmem18*) or significant (*Ahr, Acp1*) correlations. Nominally significant correlations (*p*<0.05) are displayed on the chart. For brown adipose tissue, no genes correlate highly significantly (*p*<0.01) with activity.

While the movement parameters mapped to several suggestive and two significant loci, the significant locus on chromosome 12 was the most striking and consistent (Figure 2A), thus we prioritized it for validation. To establish the effect of the Chr12 locus, we examined a congenic strain of B6 with the D2 locus at the region of interest (B6.D2N-*Ahr^d^*) [16]. We sequenced this line and observed it carries a 6 Mb segment of D2 genome on Chr 12 between 34.60 and 40.48 Mb, while the rest of the genome is B6 (though several dozen individual SNPs—i.e. spontaneous mutations—are observed elsewhere in the genome, see Materials & Methods). Ten males from all three cohorts were then entered into the same phenotyping platform until 23 weeks of age, at which point the movement experiment was performed. As predicted, the congenic line and D2 moved significantly more than the B6 animals, while the congenic line and D2 moved the same amount (Figure 2B), validating the QTL as causative of movement variance. Moreover, these increases matched the calculated effect size from the QTL: ambulatory activity increased by 1500 counts/hr, while rearing activity increased by 380 counts/hr. This analysis validated the QTL as influencing movement, though did not indicate which candidate gene(s) cause the effect (Figure 2C). The broad-sense QTL, from 30.3 to 37.6 Mb, contains 38 genes, including 9 which are under the narrow-sense QTL (35.5 to 37.6 Mb). The congenic region, from 34.6–40.5 Mb, contains 17 genes, 13 of which are within the QTL bounds. We retained all 42 genes (38 within the QTL, 4 exclusively in the congenic region) for subsequent bioinformatic analyses, though with a particular eye for the 9 genes overlapping in the congenic and significant narrow-sense QTL.

### Selecting the QTG

To select candidate genes for validation experiments, we used several established methods to prioritize candidate gene(s), which are most likely to influence movement [19]. First, the sequence variants were examined for all candidate genes, including within 5 kb of the 3′ or 5′ untranslated regions. 10 of the candidates are identical by descent across all of the BXD strains, making these genes unlikely to be causal for the QTL [20], including 3 of the 9 priority candidates. For the 32 genes with sequence variants, seven have protein-coding changes: peroxidasin homolog (*Pxdn*), thyroid peroxidase (*Tpo*), histone deacetylase 9 (*Hdac9*), mesenchyme homeobox 2 (*Meox2*), transmembrane proteins 18 and 195 (*Tmem18* and *Tmem195*), and *Ahr*. To further rank the candidate genes, we examined the transcriptional variance and regulation in eight diverse microarray datasets. Candidate genes with higher transcript variability, which have strong genetic variants at the same locus as the phenotype, and/or which associate with the phenotype are more likely to be the QTG under the QTL [19].

First, we examined the 42 genes in 8 datasets from 6 tissues. In three tissues—liver, brown adipose (BAT), and quadriceps—measurements were performed in the same BXD animals, which mapped to the movement QTL. Quadriceps and liver were also sampled in the same BXD strains on a high fat diet. The other three tissues—hypothalamus, pituitary, and adrenal—were collected previously and published by other research groups in the same BXD strains in similar conditions (i.e. age, sex, diet) [21]. All transcripts were detected in at least one tissue except *Slc26a3* (in the congenic interval) and *Prps1l1* (in the narrow QTL region). We then mapped all transcripts to identify the existence and location of significant expression QTLs (eQTLs; LRS≥20, Figure 2D). No *trans*-eQTLs were consistent across more than one dataset, while four genes gave consistent significant *cis*-eQTLs: acid phosphatase 1 (*Acp1*, in 5 datasets), *Tmem18* (in 4), *Ahr* (in 3) and syntrophin gamma 2 (*Sntg2*, in 3). We then examined the transcript variance of all candidate genes in the eight datasets. Most genes had at significant variability across the strains in each tissue (50% have variance ≥1.75 fold; Figure 2E), with three genes, *Sh3yl1*, *Prkar2b* and *Acp1*, being particularly highly variable (range ≥3.0 fold) in multiple tissues. However, most genes did not covary across the tissues, with only two having particularly consistent expression: *Ahr* and *Acp1* (Figure 2F). We last examined how the expression of each gene associated with movement phenotypes in the BXDs, with particular focus on candidates under the significant QTL and congenic locus (e.g. *Ahr*, *Sostdc1*, *Ispd*) and those with major or consistent transcript variance (e.g. *Acp1*, *Prkar2b*, and again *Ahr*). Only two genes, *Ahr* and *Acp1* had significant correlations after multiple testing correction (Figure 2G), though several other genes yielded consistent but non-significant correlations (e.g. *Tmem18*). Together, these bioinformatic analyses indicated several genes as potentially causative of movement variance, and with one top candidate: *Ahr*, which was prioritized as the first gene for validation as the quantitative trait gene (QTG), as the other strong candidate gene, *Acp1*, was not in the congenic region or under the peak QTL.

### The Effect of *Ahr* on Movement Is Highly Conserved Across Evolution

**Figure 3:**
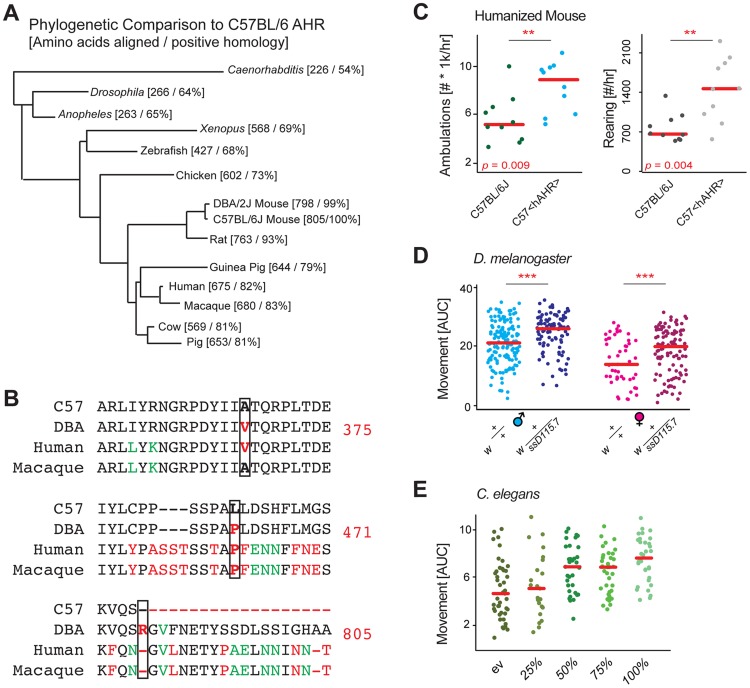
Evolutionary analysis links *Ahr* to movement. (**A**) Phylogenetic BLAST analysis of mouse *Ahr* showed that the gene is highly conserved down to simple multicellular animals such as *C. elegans*, the gene likely has conserved basic metabolic functions. (**B**) Sequence analysis of the three missense mutations of *Ahr* between B6 and D2 known to have an impact oh AHR activity (375, 471, 805). (**C**) B6 mice with the humanized AHR allele are nearly twice as active as controls. The humanized AHR allele is similar to the D2 allele in many tests of enzymatic activity, with a ∼90%+ reduction in activity compared to the B6 allele [24]. (**D**) *D. melanogaster* with a heterozygous deletion allele of the *Ahr* ortholog *ss* are also significantly more active than controls. The 50% reduced expression appears to increase movement by about 20% in both males and females. Each comparison is a separate Welch's *t*-test with *p*<0.001. Females are ∼30% less active than males in both instances (*p*<0.001). (**E**) *C. elegans* treated from early development with RNAi for *ahr-1* are nearly twice as active as worms treated with a control vector. Reduced doses of RNAi have intermediary effects on activity. *p* = 2.9e-6 for 100% vs. empty vector (ev).

*Ahr* is strongly conserved throughout evolution (Figure 3A), and acts as a bHLH-type transcription factor with impact on development and homeostasis in all species [22]. Given this consistency, we examined whether movement regulation may be another conserved physiological process regulated by the gene. In the BXDs, three particular SNPs have been established as causal for differences in *Ahr* by affecting its enzymatic activity (A375V), ligand binding and the *cis*-regulatory mechanism (L471P), and protein length (*805R, which adds 43 amino acids to the C terminus) [18], [23]. Strikingly, these three particular variants are conserved in humans, with the most common human allele (*hAHR*) humans matching the D2 allele at all three (Figure 3B) [24]. Correspondingly, *hAHR* enzymatic activity is similar to that of D2 mice [24], [25]. Moreover, the valine at position 375 (or 381 in humans) is unique among mammalian reference genomes to D2 and humans, and is not even found in macaques, which likewise have much higher *Ahr* activity than humans and D2-type mice [26], [27]. To examine a potential link between *hAHR* and activity, we phenotyped transgenic B6 mice with the *hAHR* allele replacing the murine *Ahr* [28]. In the same home cage monitoring experiments as those used before, we observed that the humanized animals were significantly more active than their wildtype counterparts (Figure 3C), and again with an increase equal to that of the congenic line (Figure 2A). This finding indicates both that *Ahr* was properly selected as the QTG, and that the effect may well be conserved cross species. To validate this hypothesis, we looked to lower organisms.

The key transcription factor motifs in *Ahr* (bHLH and PAS) are highly conserved in the *D. melanogaster* ortholog called *spineless* (*ss*) [29], and the *C. elegans* homolog called *ahr-1* [30], thus we hypothesized the regulation of movement may be further conserved to these simpler model organisms. We first examined movement in *D. melanogaster*, where we crossed the *w* strain with a loss of function allele *ss^D115.7^* [29], and examined this line as a heterozygous knockout, both in males and females. In both models, *ss* reduction resulted in a robust ∼25% increase in movement (Figure 3D). Given the conservation to *D. melanogaster*, we hypothesized that this connection may manifest also in *C. elegans*. As before, inhibition of *ahr-1* by RNAi resulted in a marked and robust increase in activity (Figure 3E). Moreover, the data in *C. elegans* indicates that the effect of *Ahr* inhibition on activity is approximately linear, at least within the expression variation tested. Full knockouts in mice, while viable, have poor postnatal survival rates [31] and have dramatically smaller livers (<50% size [32]); likewise, full knockouts in *Drosophila* of *ss* have notable morphological problems [22].

**Figure 4:**
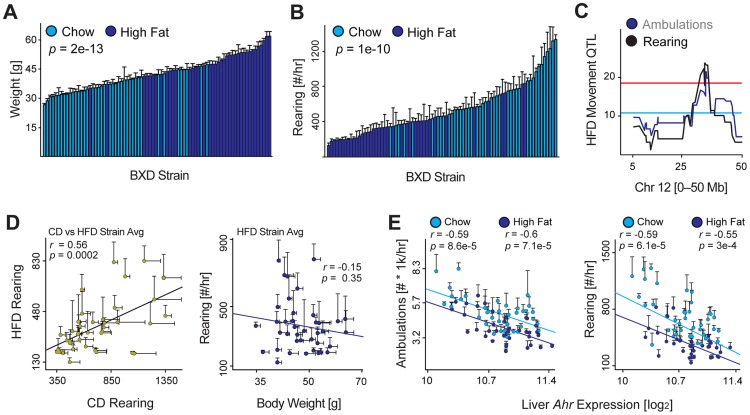
Environmental effects on movement and *Ahr*. (**A**) Weight is significantly increased in cohorts fed HFD (dark blue) by 23 weeks of age—after 15 weeks on HFD—compared to the CD-fed cohorts (light blue). (**B**) Rearing activity and ambulatory activity are significantly reduced by HFD feeding. (**C**) Both movement parameters also map significantly to the *Ahr* locus in BXDs fed HFD, indicating that the effect of HFD on movement is independent of this locus. (**D**) Left: Rearing and movement activity in HFD cohorts correlates well with the movement of chow diet (CD) cohorts run previously. Right: Body weight has no effect on spontaneous activity in mice on HFD, which is similar as observed previously for animals on CD. (**E**) Ambulatory activity (left) and rearing activity (right) correlations with *Ahr* expression in CD and HFD BXD cohorts. *Ahr* expression is unaffected by HFD.

*Ahr* inhibition robustly increased movement in all models examined, and the effect was observed in the absence of any clearly established *Ahr* ligand. In *Drosophila* and *C. elegans*, the *Ahr* orthologs are suspected to be exclusively constitutively active [33]. However, this does not indicate whether it is constitutive activity operating in the mice, or if it is an unknown dietary component of the chow diet common to all cohorts, which could influence movement in a ligand-dependent manner. As known *Ahr* ligands are either known to be non-selective and activate multiple signaling pathways (e.g. resveratrol or quercetin [34], [35]) or are highly toxic and poorly suited for normal physiological studies (i.e. TCDD), we chose to expose BXD cohorts to an environmental component that is known to influence movement: a high fat diet (HFD). We raised males from the same BXD strains, but now on a HFD from 8 until 23 weeks of age at which point we again measured spontaneous activity (Figure 4A). HFD robustly increases body weight by this age, by about 12 grams or ∼35% of body weight in each strain (Figure 4A). Movement is similarly affected, with rearing decreasing by ∼50% (Figure 4B) and ambulatory movement by ∼25%. Strikingly, both movement parameters again map to the same locus on Chromosome 12, indicating the genetic effect of this locus is independent of the dietary influence on movement (Figure 4C). Moreover, the dietary effect is consistent across all strains (Figure 4D, left) and is not directly due to the increase in body weight (Figure 4D, right). Thus, while there may be a gene-by-environment effect of diet on movement in the BXDs, it is independent of *Ahr*, which is equally expressed in both dietary cohorts. As in the CD cohorts, *Ahr* expression has a strong negative correlation with spontaneous activity in the HFD population (Figure 4E). This independent phenotype both confirms the locus identified using CD cohorts, but also indicates that *Ahr* can influence movement in mammals independently of incidental dietary or environmental effects.

## Discussion

In this study we characterized 43 strains in the BXDs genetic reference population to assess basal physiological parameters: movement and body weight. Both phenotypes are driven by numerous and complex interactions between genes and environment, and also by neurological/motivational states and by physiological limitations. In the BXDs, body weight and activity are sexually dimorphic, highly variable, and heritable. Surprisingly, body weight and food intake have no impact on standard spontaneous activity in either CD or HFD-fed cohorts despite a major decrease in movement in HFD cohorts. In both dietary groups, we identified a single common QTL causal of ∼25–40% of the variance of movement across the population. Using a congenic line, we confirmed the effect of this locus and set out to establish the causal gene through a bioinformatics approach. By analyzing nine diverse tissues, five in the cohorts phenotyped and three from other published BXD studies, we were able to establish the aryl hydrocarbon receptor (*Ahr*) as the single best candidate gene for mechanistic validation.

*Ahr* is an evolutionary conserved transcription factor involved in development, signal transduction, and metabolism [36], [37]. *Ahr* has constitutive activity, but can also be activated by a variety of ligands such as the endogenous metabolite, kynurenine, and a wide variety of environmental chemicals. Moderate to strong (∼95%) reductions in *Ahr* activity appear to have little negative effect an organism's health or viability, whether in D2 or humanized hAHR mice, or in heterozygotic fly mutants. Through cross-species analysis to *Drosophila* and *C. elegans*, we were able to confirm that reducing the expression of this gene consistently leads to an evolutionarily consistent increase in spontaneous movement.

After examining the same BXD strains on a HFD, we observed that despite a major decrease in movement in this population, the *Ahr* QTL is consistent and with a similar effect size, independently of this major environmental perturbation. This conserved effect in BXDs across different environmental conditions indicates a constitutive role for *Ahr* in the regulation of movement in vertebrates as well. The observation that reduction of *Ahr* orthologs in invertebrates has a consistent effect on movement furthers this hypothesis, as the *Drosophila* ortholog (*spineless*) is constitutively active [38] and does not appear to be affected by any exogenous ligands [33]. A large number of *AHR* polymorphisms have been identified in large and diverse human population studies [39], [40], though it remains to be seen if these variants lead to variation in locomotion and/or disposition to exercise in humans as in mice. However, as the movement link is conserved in mice with a humanized *Ahr* allele, it seems likely that natural variation in *Ahr* or of its ligands may explain part of the natural variation in human proclivity for activity. Furthermore, while our data indicate constitutive *Ahr* activation as a regulator of movement, it is conceivable that this role may be further modulated by specific ligands in mice and humans. In combination, our study expands the phenotypic roles of AHR, endowing it with a commanding role in the control of movement that is conserved across evolution.

## Methods

### Animals

All animals were communally housed by strain until phenotyping and fed a chow diet (CD; (Harlan 2018; 6% kCal/fat, 20% kCal/protein, 74% kCal/carbohydrate) throughout life after weaning. All BXD strains (BXD43–103) were originally sourced from the vivarium at the University of Tennessee Health Science Center (Memphis, TN, USA) then bred for two or more generations until progeny entered the phenotyping colony.

Male versus Female Phenotyping: 136 retired breeders (68 female, 68 male) from 22 strains (male) or 19 strains (female; all 19 overlap) were taken at 20±4 weeks of age from a breeding colony at the EPFL facility and transferred to the phenotyping unit. Males and females were separated for 3+ weeks to ensure pregnant females were not phenotyped. Males and females were phenotyped on separate days, with 10–16 animals entered into the phenotyping program every 2 days.

CD Male Phenotyping: 196 male mice from 43 strains of the BXD family were bred at the EPFL facility and transferred to the phenotyping unit at 8 weeks of age. Each cohort was communally housed (3–5 animals per cage) under 12 h light, 12 h dark cycle with *ad libitum* access to food and water at all times. Animals were in solitary cages only for the movement phenotyping test (48 hours) and were sacrificed 5 weeks after at ∼28 weeks of age.

HFD Male Phenotyping: 186 animals from 42 strains (all but 1 overlapping) were entered into the colony as before, with HFD starting at 8 weeks of age (Harlan 06414; 60% kCal/fat, 20% kCal/protein, 20% kCal/carbohydrate). Animals were in solitary cages only for the movement phenotyping test (48 hours) and were sacrificed 5 weeks after at ∼28 weeks of age.

Congenic AHR mice were purchased from The Jackson Laboratory (stock number 002921), with animals delivered at 8 weeks of age along with control B6 and control D2 mice. The congenic mice were generated by crossing B6 with D2, followed by successive backcrossing (N13) to return the B6 genome except in the region of *Ahr* [16]. The congenic region was genotyped independently to confirm the size of the interval. We then sequenced the DNA of the congenic strain using an Ion Proton PI Chip at 1.5× depth (i.e. ∼15 million 200 bp reads) and aligned it against the C57BL/6J reference. We confirmed the reported congenic interval is the only region that retains a D2 background. Several dozen SNPs were observed throughout the genome outside the reported Chr12 interval (∼34.6 to 40.5), but were distributed evenly across the chromosomes and consequently represent spontaneous mutations or sequencing and assembly errors, rather than residual D2 genotype. 10 humanized AHR mice were ordered from Taconic (model 9165), delivered at 8 weeks of age along with B6 controls [25]. The transgenic mice have exons 3–11 (of 11) replaced with hAHR, while exons 1–2 retain the B6 sequence, retaining 2 amino acids unique to B6 not present in hAHR. The key mutations (375, 471, and the lost stop codon) are present in the transgenic animal.

For tissue collection on CD and HFD BXD cohorts, animals were sacrificed under isoflurane anesthesia and cardiac perfusion after an overnight fast. High fat diet treatment and two day isolation for the recording experiment were considered as having low impact on the animals' welfare, while all other measurements and conditions were considered as having no negative impact. All research was approved by the Swiss cantonal veterinary authorities of Vaud under licenses 2257.0 and 2257.1.

### Movement Phenotyping Tests

Home cage monitoring was performed at 23±1 weeks of age for all mice except retired breeders (23±4 weeks), using a laser detection grid developed by TSE Systems (Bad Homburg, Germany) and used in the animals' standard housing cages. The detection grid has two layers: one for detecting X-Y movement (“ambulations”) the other for Z movement (“rearings”). Both measurements are technically independent, though the measurements of movement are strongly correlated (*r*∼0.70, see Figure 1B). Animals were housed individually for the 48-hour experiment starting at about 10am, with the night cycles (7pm–7am with 30 minutes of both dawn and dusk) used for movement calculations [41].

*D. melanogaster* lines containing a null mutation in the *spineless* gene, the *D. melanogaster* ortholog of *Ahr*, *w; ss^D115.7^/TM3*, were obtained courtesy of Ian Duncan's laboratory and passaged for two generations in a standard incubator. This line was crossed with w^−^ and the progeny segregated accordingly (i.e. *w* vs. *w;^ssD115.7^* and *w;TM3, hb-LacZ* vs. *w^ssD115.7^/TM3, hb-LacZ*). Movement was recorded by placing flies in a sealed chamber, tapping the chamber, and recording their movement as they naturally climb towards the top. For the tapping test, 1–2 day old flies were recorded using a standard SLR camera with a Leica macro objective. The experiment was performed four times for each cohort with one minute recordings each, with a “tap” sending the flies to the bottom of the chamber every 10 seconds. The speed with which flies reached the top of the chamber was measured using the Parallel Worm Tracker for MATLAB, which we modified slightly to work with *D. melanogaster* [42]. This speed was converted into distance by taking the area-under-the-curve (AUC) integral of their velocity.

*C. elegans* movement was recorded for 45 seconds at days 2, 3, and 4 of adulthood using a Nikon DS-L2/DS-Fi1 camera and controller setup, attached to a computerized Nikon bright field microscope. Seven plates of worms, with 10 worms per plate, were measured in each condition. The movement of worms during this time was calculated by following the worm centroids using the same modified version of the freely-available for the Parallel Worm Tracker as above.

### Bioinformatics and Statistical Analysis

R was used for basic analysis of phenotypic data. GeneNetwork (www.genenetwork.org) was used for correlation and genetic analyses. The original phenotypes published in this paper and all microarray data generated in these cohorts are available for public analysis or download using the GeneNetwork database (Species: Mouse, Group: BXD, Type: Adipose mRNA, Liver mRNA, or Muscle mRNA, then select the EPFL datasets). The three historical BXD mRNA datasets, for adrenals, pituitary, and hypothalamus, are also available here [43].

Phenotype data were checked for normality using the Shapiro-Wilk test, with a W-value ≥0.80 accepted as approximately normal. Heritability was calculated by one-way ANOVA—the aov() function in R—taking the sum of squares of within-strain variance divided by the total sum of squares variance. Dot plots are represented as individual measurements, or mean+SEM depending on the figure panel. Dot plots with error bars (e.g. Figure 1D) indicate each dot is a strain average of ∼5 individuals. Individual QTL plots consider a suggestive LRS≥12 and significant LRS≥18. Large scale QTL plots (Figure 2D) use LRS≥20 for significance due to multiple testing. Welch's *t*-tests were performed for two-way comparisons between phenotype data, as variances were typically unequal in these comparison groups. Student's *t*-tests were performed for array data, as all data are normally distributed with equal variance. Pearson's *r* is calculated for correlation plots as no outliers were observed. A *p*-value of less than 0.05 was considered the significance threshold for all analyses, except in QTL mapping when correction for multiple testing was used. All BXD phenotype data can be found on GeneNetwork.org under the “Type: Phenotype” entry then by searching for “Lisp3”.
